# Case Report: FOLFOX-induced vulvar mucositis: a rare side effect of 5-FU

**DOI:** 10.3389/fonc.2026.1824804

**Published:** 2026-06-22

**Authors:** Magali de Sauvage, Jeffrey Patterson-Fortin

**Affiliations:** Division of Oncology, Huntsman Cancer Institute, University of Utah, Salt Lake City, UT, United States

**Keywords:** 5-Fu (5-Fluorouracil), adverse event, mucositis, rectal cancer, side effect, vulvar mucous membrane

## Abstract

Acute vulvar mucocutaneous reactions during fluoropyrimidine-based chemotherapy are extremely rare. We describe the case of a 53-year-old woman diagnosed with stage IIA rectal adenocarcinoma undergoing neoadjuvant modified FOLFOX-6 (mFOLFOX-6) chemotherapy who developed an acute vulvar mucocutaneous reaction after one cycle of chemotherapy. Treatment was initiated with low-potency topical steroid cream without resolution and progressive pain requiring treatment intensification with high-potency topical steroid cream, topical lidocaine, and topical vaginal estradiol. Given the temporal association with chemotherapy and clinical features suggestive of a drug-related mucocutaneous reaction, possibly consistent with fixed drug eruption, the 5-fluorouracil (5-FU) bolus was omitted from subsequent cycles, allowing completion of all rounds of planned neoadjuvant chemotherapy without recurrence. This case expands the spectrum of known mucocutaneous toxicities associated with fluoropyrimidine-based chemotherapy and demonstrates that the omission of the 5-FU bolus preserves treatment efficacy while preventing the recurrence of this rare toxicity.

## Introduction

Rectal cancer accounts for approximately one-third of colorectal malignancies, representing approximately 46,000 new cases diagnosed annually in the United States (1). Most rectal cancers are diagnosed after a patient presents with lower gastrointestinal hemorrhage or undergoes routine screening colonoscopy. Following histological diagnosis, staging using both computed tomography (CT) of the chest, abdomen, and pelvis, and pelvic magnetic resonance imaging (MRI) is recommended (National Comprehensive Cancer Network (NCCN) Guidelines) to delineate non-metastatic and metastatic disease. Non-metastatic rectal adenocarcinoma is typically treated using a multi-modality approach with chemotherapy, radiotherapy, and surgery. Regarding chemotherapy, 5-fluorouracil (5-FU) and oxaliplatin (FOLFOX) are the most studied and effective regimens based on several recent phase III clinical trials in rectal cancer (RAPIDO, PRODIGE 23, OPRA, and PROSPECT) (2–6). The PROSPECT trial demonstrated that neoadjuvant chemotherapy with selective, response-guided use of chemoradiation therapy (CRT) achieved similar disease-free survival (DFS) and overall survival (OS) compared to neoadjuvant CRT alone, with approximately 90% of patients avoiding radiation and its associated late toxicities (7).

While FOLFOX is a well-characterized chemotherapy regimen with peripheral neuropathy as its dose-limiting toxicity, mucocutaneous adverse effects, including oral mucositis and stomatitis, are well recognized ( (7); FDA Drug labels for fluorouracil and oxaliplatin). Mucositis occurs more frequently with bolus 5-FU administration compared to continuous infusion, and the addition of oxaliplatin to fluoropyrimidine-based regimens modestly increases the incidence of mucositis/stomatitis compared to 5-FU alone (37% vs 32% all grades in previously treated patients) (FDA Drug labels for fluorouracil and oxaliplatin). However, vulvar mucocutaneous involvement during FOLFOX chemotherapy is exceedingly rare and has not been well described in the literature.

Notably, women experience 5-FU-induced mucosal toxicity more frequently and with greater severity than men. In a Southwest Oncology Group analysis, the incidence of stomatitis was 63% in women versus 52% in men, with severe or very severe stomatitis occurring in 22% of women compared to 12% of men (8). Similar gender-based disparities have been observed across multiple 5-FU toxicities, including leukopenia (9). Whether vulvar mucocutaneous reactions are underdiagnosed or underreported, given the intimate nature of symptoms and potential patient reluctance to disclose, remains unknown but warrants consideration. Our case represents an acute vulvar mucocutaneous reaction temporally associated with neoadjuvant modified FOLFOX-6 (mFOLFOX-6) chemotherapy, highlighting a rare but clinically significant toxicity that may disproportionately affect women.

## Case presentation

A 53-year-old woman with a past medical history notable for a seizure disorder well controlled by lamotrigine presented to her primary care physician with alternating constipation and diarrhea, and daily rectal bleeding for several months. Esophagogastroduodenoscopy (EGD) was unrevealing with a normal esophagus, normal stomach, normal duodenum, and a small hiatal hernia. Colonoscopy demonstrated a fungating, infiltrative, ulcerated non-obstructing mass at the rectosigmoid junction, approximately 20 cm from the anal verge and 5 cm in length. Pathology confirmed invasive adenocarcinoma. Staging CT chest, abdomen, and pelvis showed no distant metastasis. Pelvic MRI demonstrated T3B N0 disease, with the tumor located in the distal sigmoid colon extending near the proximal rectum. The patient established care with Medical Oncology, Radiation Oncology, and Surgical Oncology. Given stage IIA disease and eligibility for sphincter-sparing surgery, the patient elected to initiate neoadjuvant chemotherapy with mFOLFOX-6 followed by selective, response-guided CRT per the PROSPECT trial, and the patient stated a goal of avoiding any late radiation-associated toxicities.

Neoadjuvant treatment was initiated with 5-FU and oxaliplatin based on the PROSPECT trial (6). FOLFOX was dosed as follows: oxaliplatin 85 mg/m^2^ intravenous (IV), leucovorin 400 mg/m^2^ IV, 5-FU 400 mg/m^2^ IV bolus, and 5-FU 1,200 mg·m^−2^·day^−1^ over 48 hours IV, every 2 weeks. Two days after the first cycle, the patient developed severe, progressive, and constant vulvar burning pain that was exacerbated by urination. Emergency department (ED) evaluation revealed erythema on the posterior aspect of the left labia majora and minora without frank ulceration. Laboratory studies were unrevealing. The patient was diagnosed with suspected vulvovaginal mucositis without full-thickness involvement and was discharged with the following recommendations from the ED:

- gentle cleansing using warm water or saline solution, avoiding soaps or products with alcohol, fragrance, or other irritants;
- topical treatment with low-potency steroid creams (1% hydrocortisone) to reduce inflammation, applied two to three times daily for 7–10 days;
- systemic pain control with acetaminophen or non-steroidal anti-inflammatories; and
- maintenance of good hydration and nutrition.

Two days later, the patient presented to the Oncology Acute Care Clinic with worsening vulvar pain exacerbated by urination. She reported decreased oral intake to avoid urination. Physical examination demonstrated bilateral erythema and skin erosion (left greater than right). Half of a speculum was used to better visualize the vagina, revealing clear demarcation with normal tissue beginning at the vaginal introitus. Laboratory studies demonstrated a pre-renal acute kidney injury (creatinine of 2.59 mg/dL from baseline of 0.9 mg/dL; blood urea nitrogen of 41 mg/dL from baseline of ~20 mg/dL), likely secondary to decreased oral intake to avoid urination and subsequent pain exacerbation. A biopsy was not performed due to significant patient discomfort and concern for additional morbidity. Gynecology was consulted.

### Differential diagnosis

The differential diagnosis of acute vulvar erosions and erythema included the following: a drug-related mucocutaneous reaction (including fixed drug eruption), severe cutaneous adverse drug reactions [Stevens–Johnson syndrome (SJS) and toxic epidermal necrolysis (TEN)], inflammatory dermatoses (lichen planus, lichen sclerosis, contact dermatitis, and psoriasis), and infectious causes (herpes simplex virus and candidiasis).

SJS and TEN were considered given the patient’s long-term use of lamotrigine, a known causative agent. However, several factors make these less likely: no recent changes in lamotrigine dosage; absence of oral, ocular, or vaginal mucosal involvement and no epidermal detachment (which occurs in over 80% of SJS cases); rapid onset of 2 days post-chemotherapy, in comparison to the typical 4–28 days for SJS; and a localized rather than widespread distribution (10).

Inflammatory dermatoses were less likely given the acute nature of symptom onset following chemotherapy initiation, whereas lichen sclerosis and lichen planus are chronic conditions that develop over months to years. Additionally, characteristic physical examination findings of lichen planus showed well-demarcated bright-red vestibular patches with Wickham striae, and lichen sclerosis presents with white, thin plaques in a characteristic figure-eight configuration involving the vulva, perineum, and perianal region with severe pruritus rather than burning pain (11). Formal microbiology testing was not performed in this case given low clinical suspicion; however, we acknowledge this is a limitation and recommend that future cases include appropriate infectious work-up to exclude these diagnoses. Contact dermatitis was unlikely given the acute onset and lack of new topical exposures.

Infectious etiologies were as follows: lower on the differential given lesion morphology, lack of associated symptoms, and patient history. Herpes simplex virus presents as characteristic painful vesicles accompanied by systemic symptoms; the bilateral symmetric distribution is atypical for primary infection (12).

The presentation was most consistent with a drug-related mucocutaneous reaction, given the temporal relationship to chemotherapy initiation, localized mucosal involvement, and absence of systemic features. Fixed drug eruption was considered within the differential, as it characteristically involves the genital mucosa and recurs at the same anatomical site upon drug re-exposure (13–15). However, in the absence of histological confirmation via biopsy, the diagnosis of fixed drug eruption remains presumptive. As noted above, biopsy was not performed in this case due to significant patient discomfiture and concern for additional morbidity. In addition, mucositis grading and treatment based on clinical findings without the need for histopathological confirmation are well established in oncologic practice and supported by the National Cancer Institute’s Common Terminology Criteria for Adverse Events (CTCAE). The non-recurrence of symptoms after the omission of the 5-FU bolus is supportive of a drug-related etiology but does not constitute a formal rechallenge, as the patient continued to receive infusional 5-FU and oxaliplatin.

### Treatment and follow-up

The initial treatment of vulvar mucocutaneous reaction with low-potency topical steroids was unsuccessful following her ED visit, prompting the patient to return to the hospital again 2 days later. Treatment was intensified, as follows:

- Clobetasol 0.05% BID,
- topical lidocaine jelly 2% TID,
- topical vaginal estradiol cream (pea-sized amount nightly for 2 weeks after improvement to promote re-epithelialization during the healing process in this postmenopausal patient),
- sitz bath TID, and
- application of barrier ointment.

The use of topical estradiol was extrapolated from American Society of Clinical Oncology (ASCO) guidelines for the management of cancer treatment-related sexual dysfunction, which recommend vaginal moisturizers and low-dose vaginal estrogen for vulvovaginal atrophy and dyspareunia, and topical lidocaine for persistent introital pain (16). While these guidelines primarily address radiation-induced and hormonal therapy-related vulvar changes, the shared pathophysiology of mucosal injury and impaired epithelial integrity provided the rationale for this approach in our patient.

The acute kidney injury was treated with 2 L of normal saline IV with resolution, and the patient was discharged home.

Five days later, the patient presented for consideration of cycle 2 of chemotherapy. She reported resolution of the vulvar pain and that she had continued using the topical steroid (Clobetasol) but discontinued the topical lidocaine. Chemotherapy with FOLFOX was continued, as her symptoms had completely resolved. Given the suspected drug-related etiology, cycle 2 was administered with the omission of the 5-FU bolus: oxaliplatin 85 mg/m^2^ IV and 5-FU 1,200 mg·m^−2^·day^−1^ over 48 hours IV, every 2 weeks. Following cycle 2 of FOLFOX without the 5-FU bolus, the patient experienced no recurrence of symptoms and discontinued the use of the topical steroids 7 days after cycle 2 chemotherapy. The patient completed all six cycles of neoadjuvant FOLFOX without a recurrence of vulvar mucositis.

**Figure 1:**
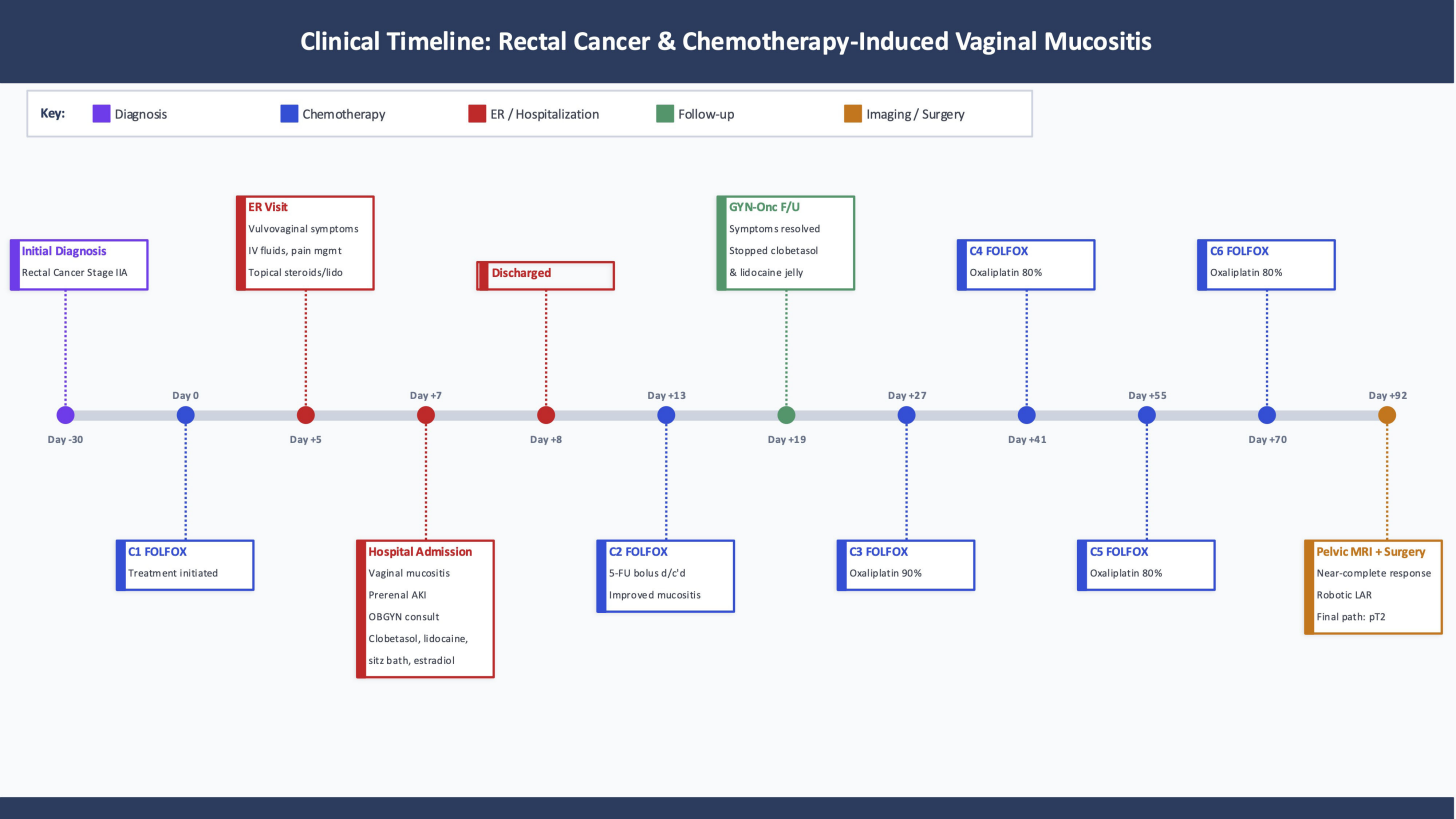
Timeline of case report.

Following the six cycles, repeat pelvic MRI demonstrated a likely complete response with marked decrease in tumor bulk, scar tissue formation, no residual tumor signal or diffusion restriction, and no enlarged lymph nodes. The patient subsequently underwent robotic lower anterior resection with final pathology demonstrating pT2 disease and treatment response (**Figure 1**).

## Discussion

The temporal relationship between mFOLFOX-6 administration and symptom onset (2 days), resolution with supportive care, and non-recurrence after the omission of the 5-FU bolus is suggestive of a drug-related etiology. However, causality cannot be definitively established. A formal causality assessment tool was not prospectively applied. Retrospective application of the Naranjo Adverse Drug Reaction Probability Scale yielded an estimated score in the “probable” range (5–8 points), based on the following: temporal relationship (+2), improvement after drug modification (+1), absence of a clear alternative etiology (+2), and partial prevention with dose modification (+1) (17, 18). However, this retrospective scoring has inherent limitations.

5-FU exerts its cytotoxic effects through the inhibition of thymidylate synthase and incorporation of fluoronucleotides into RNA and DNA, leading to impaired DNA synthesis and cell death in rapidly dividing cells (FDA Drug label for fluorouracil). Mucosal epithelial cells, which have a high mitotic rate, are particularly vulnerable to these effects. The pathogenesis of 5-FU-induced mucositis involves direct epithelial cell apoptosis, generation of reactive oxygen species, activation of inflammatory cascades including NF-κB signaling, and disruption of the epithelial barrier (19). Bolus administration of 5-FU produces higher peak plasma concentrations compared to continuous infusion, which may explain the higher incidence of mucositis observed with bolus dosing (FDA Drug label for fluorouracil).

Oxaliplatin, a third-generation platinum compound, exerts cytotoxicity through the formation of platinum–DNA adducts that inhibit DNA replication and transcription. While oxaliplatin’s primary dose-limiting toxicity is peripheral neuropathy, its combination with 5-FU modestly increases the incidence of mucosal toxicity compared to 5-FU alone (FDA Drug label for oxaliplatin). Therefore, the contribution of oxaliplatin to the mucocutaneous reaction observed in our patient cannot be excluded. Furthermore, the omission of the 5-FU bolus, while still receiving infusional 5-FU, from subsequent cycles, with non-recurrence, suggests that peak drug concentration from bolus administration may have been the precipitating factor, consistent with the known association between bolus 5-FU and higher rates of mucositis. However, this does not constitute a formal rechallenge, as the patient continued to receive infusional 5-FU.

The mucosa is the membrane that lines body cavities and the surface of internal organs and is composed of one or more layers of epithelial cells. It is frequently replenished via the activity of rapidly dividing basal epithelial cells dependent on DNA synthesis, thus rendering them vulnerable to toxicity secondary to 5-FU. Administration of 5-FU is commonly known to cause oral and gastrointestinal mucositis as a side effect secondary to epithelial thinning, loss of barrier integrity, and ulceration, leading to an inflammatory cascade with painful erythema and ulceration (20). The vulvar mucosa shares histological features with other mucosal surfaces susceptible to chemotherapy-induced injury, including a stratified squamous epithelium with high cellular turnover. However, the mechanism underlying site-specific localization to the vulva, rather than the more commonly affected oral mucosa, remains unclear. Possible contributing factors include local microenvironmental differences, friction and moisture exposure, and individual patient susceptibility. In our patient, postmenopausal mucosal atrophy may have represented an additional predisposing factor, although baseline vulvar examination prior to chemotherapy initiation was not documented. While 5-FU acute vulvar mucocutaneous reaction has not been reported in the literature, the physical examination of our patient, notably with bilateral erythema and skin erosion, is consistent with this diagnosis.

The management of vulvar mucositis is underrepresented in oncology guidelines, with the main focus of these guidelines being on the management of oral and gastrointestinal mucositis (for example (21),). Thus, the management of this case was informed by principles extrapolated from the treatment of chemotherapy- and radiation-induced mucosal toxicity in gynecologic oncology. ASCO clinical practice guidelines recommend low-dose vaginal estrogen and vaginal moisturizers for cancer treatment-related vulvovaginal symptoms and topical lidocaine for introital pain (16). While these recommendations were developed primarily for radiation-induced and endocrine therapy-related vulvovaginal changes, the underlying pathophysiology of mucosal injury, impaired epithelial regeneration, and local inflammation is shared with chemotherapy-induced mucocutaneous toxicity. The stepwise escalation from low-potency to high-potency topical steroids, combined with topical analgesia and estradiol for mucosal restoration, proved effective in our patient.

The decision to omit 5-FU bolus while continuing 5-FU infusion is supported by evidence that in the setting of metastatic/advanced gastrointestinal malignancies, the omission of the 5-FU bolus leads to reduced treatment toxicity without impacting efficacy (22). Additionally, the SCOT trial protocol specified that for FOLFOX-related toxicity, the 5-FU bolus should be omitted before reducing doses of oxaliplatin or infusional 5-FU (23). NCCN guidelines also explicitly include mFOLFOX-7 (which omits the 5-FU bolus) as an equivalent alternative to mFOLFOX-6 for both colon and rectal cancers.

In this patient, omitting the 5-FU bolus was a rational strategy to reduce the risk of recurrent mucositis, which would cause treatment delays, interruptions, or discontinuations, while preserving treatment efficacy through continued infusional 5-FU. This approach was validated by the excellent oncologic response in our patient, with likely complete pathological response on repeat pelvic MRI and downstaging to pT2 disease with treatment response on pathology.

## Limitations

This case has several important limitations that should be acknowledged. First, causality cannot be definitively established. No validated causality assessment tool (e.g., Naranjo scale) was prospectively applied, although retrospective scoring suggests a “probable” (score 6) adverse drug reaction (17, 18). Second, no biopsy was obtained to confirm the diagnosis histologically, and no microbiological testing was performed to formally exclude infectious etiologies. Regarding the lack of a biopsy, this was deferred due to significant patient discomfort, as well as the fact that it is well established in oncologic practice and supported by the CTCAE to clinically diagnose and treat mucocutaneous toxicity from 5-FU without histopathological confirmation. While clinical suspicion for infection was low, these omissions reduce the strength of causal inference. Third, the patient’s baseline vulvar condition prior to chemotherapy initiation was not documented; therefore, pre-existing vulvar conditions (e.g., mucosal atrophy and chronic dermatoses) that may have predisposed to or confounded the presentation cannot be excluded. Fourth, the diagnosis of fixed drug eruption remains presumptive without histological confirmation or formal drug rechallenge, and this entity should be considered within the differential diagnosis rather than as a confirmed diagnosis. Fifth, the reaction cannot be attributed exclusively to 5-FU, as the patient received a multi-agent regimen (mFOLFOX-6), and the contribution of oxaliplatin or drug interactions cannot be excluded. Finally, this is a single case report, and the generalizability of both the clinical presentation and the management approach, including the efficacy of 5-FU bolus omission in preventing recurrence, requires validation in additional cases.

## Summary

This case illustrates the successful identification and management of an acute vulvar mucocutaneous reaction temporally associated with mFOLFOX-6 chemotherapy. The reaction was likely related to 5-FU, particularly the bolus component, given the temporal relationship and non-recurrence after bolus omission. However, definitive causality cannot be established, and the contribution of other regimen components cannot be excluded.

For clinicians encountering similar presentations, we recommend the following practical approach:

1. Diagnostic work-up: Perform a thorough vulvar examination with photographic documentation. Obtain microbiological testing [viral culture or PCR for herpes simplex virus (HSV) and fungal culture] to exclude infectious etiologies. Consider punch biopsy for the affected area for histological confirmation, particularly to distinguish between mucositis, fixed drug eruption, and other dermatoses, if clinically appropriate. Apply a validated causality assessment tool such as the Naranjo scale (17, 18). Document vulvar status prior to chemotherapy initiation when possible.
2. Management: A stepwise approach beginning with low-potency topical steroids, escalating to high-potency steroids (Clobetasol 0.05%) and topical lidocaine for pain control, is reasonable. In postmenopausal patients, topical vaginal estradiol may promote mucosal re-epithelialization, consistent with ASCO guideline recommendations for cancer treatment-related vulvovaginal symptoms. Sitz baths and barrier ointments provide additional symptomatic relief (16).
3. Chemotherapy modification: Omission of the 5-FU bolus while continuing infusional 5-FU and oxaliplatin allowed completion of all planned neoadjuvant chemotherapy without recurrence and without apparent compromise of treatment efficacy, as evidenced by the near-complete pathological response. This approach is consistent with the known higher incidence of mucositis with bolus versus infusional 5-FU (FDA Drug label for fluorouracil).
4. Recurrence and prevention: In our patient, no recurrence was observed across five subsequent cycles after bolus omission. Whether prophylactic measures, such as preemptive topical barrier protection, mucosal moisturizers, or dose reduction rather than bolus omission, could prevent initial episodes in high-risk patients (e.g., women and postmenopausal status) remains unknown. Clinicians should counsel patients receiving fluoropyrimidine-based chemotherapy about the possibility of genital mucocutaneous reactions and encourage early reporting of symptoms.

## Learning points

- Acute vulvar mucocutaneous reactions during fluoropyrimidine-based chemotherapy are extremely rare. Clinical presentation can mimic other mucocutaneous syndromes.
- Management requires a multimodal supportive care approach: high-potency topical corticosteroids, topical anesthetics, and consideration of topical estrogen in postmenopausal patients.
- Omission of the 5-FU bolus may prevent the recurrence of mucositis without compromising oncologic efficacy.
- This case expands the spectrum of known 5-FU-associated mucosal toxicities, underscoring the need for clinician vigilance and individualized supportive interventions when managing atypical fluoropyrimidine reactions.

## Data Availability

The original contributions presented in the study are included in the article/supplementary material. Further inquiries can be directed to the corresponding author.
